# Mobile Health Technology for the Personalized Therapy of Hemophilia

**DOI:** 10.3389/fmed.2021.711973

**Published:** 2021-08-10

**Authors:** Noemi Dirzu, Ionut Hotea, Ciprian Jitaru, Melen Brinza, Laura Urian, Mareike-Catrina Peters, Krisztina Gal, Louis Popescu, Cristina Blag, Mirela Marian, Eva Pal, Marilena Stanescu, Diana Cenariu, Cristina Tarniceriu, Margit Serban, Delia Dima, Daniel Coriu, Ciprian Tomuleasa

**Affiliations:** ^1^Medfuture Research Center for Advanced Medicine, Iuliu Hatieganu University of Medicine and Pharmacy Cluj Napoca, Cluj Napoca, Romania; ^2^Department of Hematology, Iuliu Hatieganu University of Medicine and Pharmacy Cluj Napoca, Cluj Napoca, Romania; ^3^Department of Hematology, Ion Chiricuta Clinical Cancer Center, Cluj Napoca, Romania; ^4^Department of Hematology, Fundeni Clinical Institute, Bucharest, Romania; ^5^Department of Hematology, Carol Davila University of Medicine and Pharmacy Bucharest, Bucharest, Romania; ^6^Department of Pediatrics, Iuliu Hatieganu University of Medicine and Pharmacy Cluj Napoca, Cluj Napoca, Romania; ^7^Department of Hematology, Emergency Clinical Children's Hospital, Cluj Napoca, Romania; ^8^Takeda Pharmaceutical Company, Tokyo, Japan; ^9^Department of Anatomy, Grigore T. Popa University of Medicine and Pharmacy, Iasi, Romania; ^10^Department of Hematology, St. Spiridon County Clinical Emergency Hospital, Iasi, Romania; ^11^Department of Hematology, Victor Babes University of Medicine and Pharmacy Timisoara, Timisoara, Romania; ^12^European Haemophilia Treatment Center, Timisoara, Romania

**Keywords:** hemophilia, IT technology, patient-tailored monitoring, mHealth, application monitoring

## Abstract

The management of patients with hemophilia has evolved significantly since the first treatment attempts were made in the late 1930s. Since then, each new step in the treatment of patients with hemophilia has brought important advancements, as well as its unique set of challenges. Today, a patient-centered, individualized comprehensive approach is the new paradigm, moving away from the traditional “one size-fits-all” approach, to provide the best possible care for each patient with a bleeding disorder. As part of this complex task, mobile health applications might have the capacity to play an important role in reaching that goal. However, the use of new electronic technologies as part of a comprehensive treatment approach for patients with hemophilia simultaneously presents a new set of challenges that needs consideration. In the first section, currently available treatment of hemophilia patients will be revised, while in the second part the role of IT software in the treatment monitoring of hemophilia patients will be discussed.

## Introduction

Hemophilia is a group of rare bleeding disorders, recessive genetic diseases linked to the X chromosome (1, 2). Due to the lack of coagulation factors from the intrinsic pathway, patients present with prolonged bleeding after injury, easy bruising, or even spontaneous bleeding (3). These symptoms pose a great risk of permanent damage if the bleeding occurs internally, inside the joints, intramuscularly, or intracranially (4). Diagnosis is made usually in early childhood, when the first bleeding episodes occur. There are two main types of hemophilia: A (HA), caused by the deficiency of factor VIII (FVIII) and B (HB) caused by the deficiency of the Christmas factor (FIX). Acquired hemophilia appears later in life, due to the formation of antibodies against the coagulation factors (5). Even more rare are the cases of hemophilia C, due to the lack of factor XI (6), and parahemophilia, caused by the lack of factor V (7). Depending on the plasma levels, hemophilia can be mild (plasma clotting factor levels of 0.05–0.4 international units (IU)/mL), moderate (0.010.05 IU/mL), or severe (<0.01 IU/mL) (4). Suspicion rises in the presence of the symptoms or family history of bleeding disorders. Diagnosis is made by determining the levels of the coagulation factors and the levels of factor inhibitors if they are present (8). Genetic confirmation of the mutations causing the disease is available, but its costs are still prohibitive. Genetic counseling should be offered to prospective parents who have a history of bleeding disorders in the family (9). Family planification is also available, but parents must know, that nowadays, with the help of the new treatment options, hemophilia is manageable, and it is no longer a life-threatening disease, if no major treatment complications occur, like the formation of an inhibitor (10). Hemophilia is a chronic disease. To this moment it has no cure, but it can be managed with a comprehensive multidisciplinary approach using clotting factor concentrates (CFC) and/or by-pass agents, with controlled symptom and complication management. Targeted treatment options are available on-demand, when bleeding occurs, or in prophylactic form (9). Studies show that patients who receive coagulation factors or substitutes prophylactically, have fewer bleeds, fewer complications, a better mental health thus a better quality of life (11). The most important adverse event of factor replacement therapy (affecting a substantial part of treated patients—some 25 to 35% of PUPs, depending on the type of CFC, plasma-derived or recombinant) is the development of neutralizing alloantibodies against the coagulation factor. Newer therapies have emerged which use non-factor molecules instead of the recombinant or plasma-derived factors, which can by-pass the deficient factor in the coagulation cascade (12). These molecules can be used in patients who have developed antibodies against coagulation factors, with fewer administrations, with more stable plasma levels, and with subcutaneous administration (13, 14).

Hemophilia is, except for acquired hemophilia, a lifelong disease which should be addressed with a comprehensive treatment approach. The goal is to improve the patient's quality of life, reduce bleeding events with the resulting complications, days spent in hospital and subsequent health care costs. Ideally, the patient should have access to a multidisciplinary team of health care specialists: a core team consisting of hematologist, hemophilia nurse, psychosocial specialist, musculoskeletal specialist, and laboratory specialist (9). Providing access to a Hemophilia Treatment Center (HTC) is an important aspect in the management of the disease, as it enables the patient to receive appropriate treatment, adequate training and education about their condition and provides a platform for exchange between peers (15).

The cornerstones in the management of hemophilia patients are represented by treatment with appropriate medicinal products, lifestyle management, patient education, self-management, and empowerment, and, in case of complications, possibly surgical interventions (9). Replacement therapy, meaning substitution of clotting factors VIII or IX with plasma derived or recombinant clotting factor concentrates (CFCs) represents the mainstay of hemophilia treatment nowadays (16). Depending on the severity of the disease, patients may be treated with on-demand clotting factor concentrate infusions only, such as in times of surgical intervention or bleeding events or receive long-term prophylaxis. Primary prophylaxis is the current standard of care for patients with severe hemophilia but should also be considered in some patients with moderate hemophilia, with recurring bleedings. It follows a regular infusion schedule, typically 2 to 3 times a week, starting from a young age (17). In case of inhibitor development, the treatment of patients with hemophilia becomes much more complex, in terms of products used, infusion frequency, follow-up treatment or costs. In case a patient with INH undergoes an ITI protocol (immune tolerance induction), surveillance of compliance becomes key for the success of this costly therapy.

Adherence to treatment is of the utmost importance. It is thus vital for these patients to be closely monitored by their caregivers. Regular assessment of the patients should be carried out through specialized hemophilia centers (18). However, the rarity of the disease does not allow for a high number of such centers (19). The patients must travel long distances to be assessed by dedicated specialists, which poses a problem compliancy-wise. At the implementation of the treatment, assessment of factor levels must be performed to calculate the correct doses and evaluate the pharmacokinetics (PK) of the therapy. An initial PK assessment before treatment initiation is desirable but not mandatory, given that most of the patients are young children. Close monitoring of these patients is of the utmost importance since the smallest bleeding can have long term effects. To travel for hours every time is not feasible. In the last year, due to the COVID-19 pandemic, accessibility to HTC is scarcer than ever. Hospital access was until recently restricted to emergencies as every hospital visit poses a great risk to both patients and HTCs (20). Patients must be tested in advance for epidemiological reasons, which for many of them is a problem and they choose to neglect their disease (21, 22). In many countries, where there is no system of regular monitoring and assessment, these patients are lost from regular and continuous follow-up and benefit from treatment only when a major bleed occurs. Many pharmaceutical companies and HTC searched for means for closer monitoring of these patients. Most of them are children and young adults who are shown to have a positive attitude toward learning trough technology (23), electronic devices turn out to be useful tools in monitoring and altogether the managing of hemophilia patients.

## Hemophilia Monitoring Applications

This systematic review was conducted according to the Prisma guidelines. Our research question was: “How is mobile health technology used in hemophilia?” An electronic search on three databases (PubMed, Web of Science, Cochrane), as well as National Institutes of Health (NIH) and European Medicines Agency (EMA) was carried out to identify the relevant articles and products published in English or developed/under development until May 2021. The following keywords were used: mobile health technology, hemophilia, management, prophylaxis, on-demand treatment. Eligible studies were considered by those who reported the following: mobile health technology, hemophilia, diagnosis and management, cohort (prospective, retrospective), cross-sectional, case-control, randomized clinical trials. All other articles who did not mention the previous criteria were excluded. Products developed and approved/under development by the NIH/EMA were also considered. Titles and abstracts of the relevant articles were screened by two independent researchers. Full texts of articles previously obtained were assessed for inclusion. The reference lists of the relevant articles were searched for additional studies. Any other discrepancies regarding the selection of the articles were resolved by a third researcher.

In the following, the role of software-supported applications in the management of hemophilia patients will be discussed. For ease of discussion, the use of mobile phone and web-based applications (apps) and software in the management of disease will be referred to collectively as mobile health (mHealth) (24). The World Health Organization (WHO) has defined mHealth as “medical and public health practice supported by mobile devices, such as mobile phones, patient monitoring devices, personal digital assistants (PDAs), and other wireless devices” (25), while the National Institutes of Health (NIH) has defined the term as “the use of mobile and wireless devices (cell phones, tablets, etc.) to improve health outcomes, health care services, and health research” (26). Von Willebrand disease in the context of mHealth is embraced as are the two types of hemophilia.

There are several applications available (Table 1), which can bring closer patients to their HTC, thus making hemophilia a much more manageable disease. Mostly, these applications help the patient to keep an accurate evidence of their infusions and symptoms and make it easy to communicate with their specialist, who in many cases is located miles away (65). With the help of mobile apps, bleeds are more promptly reported and treated (58). Many of these apps offer educational materials which is a great resource not only for the patients, but for the local caregivers who are in closer contact, but not always well-prepared to deal with this kind of patients. Still, such educational material needs to be validated by competent individuals or institutions and serve exclusively the purpose to optimize the treatment prescribed by the hemophilia specialist. It is very important for the patients and their families to know what they are dealing with, to be part of a community who understands their struggles. As hemophilia is a rare disease, patient communities have a hard time to physically gather and share their experience. Some of the applications have a social media structure, helping people who are struggling with these disorders to connect and form a network. Lifestyle changes and physical activity monitoring can be also carried out with the help of these apps (46). Also, it is important for traveling patients to be able to locate the HTC closest to their location. For the apps listed and described in Table 1, we did not find any information or statements on who owns the collected data or who has the overall control over the data. Thus, data ownership needs to be described *expressis verbis* in the written informed consent by those who use any of the apps.

**Table 1:** mHealth technologies for hemophilia monitoring.

**App**	**Developer**	**Availability/Scientific articles**	**Age**	**Language**	**Function**	**Addressability**
MicroHealth Hemophilia	MicroHealth Inc.	Android (27)Apple (28)	17+	EnglishSpanish	Logging of infusions (Product, date, time, site, dose, type, lot number, expiration date, reminders)Factor inventory levelsTracking of bleeds (date, time, severity, reason, treatment)—photoActivity tracker EducationNetworking community	Patients, Doctors, Pharmacists
myPKFiT	Takeda Pharmaceuticals	Android (29)Apple (30)Browser/(31, 32)	16+	English	Logging of infusions (Date, time, dose, reason, site, lot number, calendar, reminders)Dose calculatorTracking of bleeds (type, date, time, site, pain level, treatment)—pinpoint, photoSpecial features for Advate® users. Estimates FVIII concentration levels by preparing an individualpharmacokinetic profile of the patient after two or more determinations of FVIII levels.Data export	Patients, Doctors
myWAPPS	Design2Code Inc.	Android (33)Apple (34)/(32, 35)	17+	EnglishKoreanCzechFinnishGermanItalianSpanishFrenchHungarianPortuguese	Logging of infusions (Date, time, dose, reason, site, lot number, calendar)Estimating the factor concentration levels by pharmacokinetic tailored studiesReminders for infusionsAlerts when concentrations get critical	Patients, Doctors
Zero Bleeds	Takeda Pharmaceuticals	Android (36)Apple (37)	12+	English	Logging of infusions (Product, date, time, site, dose, type, lot number, alerts, reminders). Tracking of symptoms (Bleeds: localization, date, time, reason, pain level, treatment) - pinpoint and photo;Compliance monitoring. Data is encrypted.	Patients, Doctors, HTCs
Hemophilia Connect	F. Hoffmann-La Roche	Android (38)Apple (39)	4+	English, French	Logging of infusions (Product, date, time, site, dose, type, lot number, expiration date, dose reminders)Tracking of bleeds (date, time, type, treatment, pain level)Tracking of physical activityEducationGamification for pediatric users Networking	Patients, Doctors
MyFactor	BioRX	Android (40)Apple (41)	12+	English	Logging of infusions (Product, date, time, dose, type, lot number, expiration date, reminders) Tracking of bleeds (pain episodes)—photo	Patients, Doctors
HemoTrax	CSL Behring LLC	Android (42)Apple (43)	12+	English	Logging of infusions (Product, date, time, site, dose, type, lot nr, expiration date, reminders) Tracking of bleeds (localization, treatment)—pinpoint, photo	Patients, Doctors
HemMobile	Pfizer Inc.	Android (44)Apple (45)/(46, 47)	4+	English	Logging of infusions (Product, date, time, site, dose, reason, lot nr, expiration date, reminders) Tracking of bleeds, pain episodes—map, photo Tracking of physical and daily activities (heart rate, steps)Ordering factorLocalization of nearest HTC	Patients, Doctors
HemoLab	Sahil Bhaldar	Android (48)		English	Logging of infusions (Product, date, time, dose, lot nr)Tracking of bleeds (date, time, cause, severity, pain level, treatmentEducation	Patients
Hemophilia Initiative	Health Initiative	Android (49)Only in testing			Logging of infusions (Product, date, dose, purpose lot nr, expiration date, reminders) Dosage calculatorTracking of bleeds (localization)Localization of nearest HTC Support—physiotherapyEducation	Patients, Doctors
Haemophilia Pal	Haemophilia Pal	Android (50)		EnglishMalay	Logging of infusions (Product, date, time, site, dose, reason, lot nr, expiration date)Dosage calculatorTracking of bleeds (localization) Education	
FactorTrack	Bayer Healthcare Anupam Godbole; Intouch Solutions, USA	My health apps (51)/(47)		EnglishItalianJapanese	Logging of infusions (Product, date, time, dose, lot number)Record of bleeds (type, reason, localization—body diagram)Education	
HemoTool	Dakota Rosenfelt, Click&Go App Design Inc.	Android (52)Apple (53)	12+	English	Logging of infusions (Product, date, time, site, dose, reason, lot nr, expiration date, reminders) Dosage calculatorTracking of bleeds, pain episodes)—body map, photoEducationLocalization of nearest HTCOrders of medication and supplies	Patients, Doctors
HIRT?	JoAnn Nilson	Android (54)		EnglishFrench	Symptom severity recognition First aid	Patients, HTCs
Haemoassist® 2—Germany	StatConsult	Apple (55, 56)/(57–59)	12+	GermanSpanish	Logging of infusions (Product, date, time, site, dose, reason, lot nr, expiration date, reminders) Tracking of bleeds	Patient, Doctor
Kibo	Hoffmann-La Roche	Android (60)		Spanish	Treatment adherence (infusions, stock)	Patients, Doctors
HemAPP	Heliant d.o.o. Serbian Society for Hemophilia	Android (61)Apple (62)	12+	Serbian	Treatment (Product, date, time, dose, reason, calendar)Bleads (date, intensity, treatment, localization)ExportMore than 5,000 users	Patients
Registro Mexicano de Coagulopatías	Federación de Hemofilia de la República Mexicana	Android (63)		Spanish	Logging of infusions (Product, Date, time, site, Tracking of bleeds, articular function, pain episodes (intensity, site, cause, treatment) EducationEstablishing a national register	Patient, Doctor
Hemofilia Indonesia	Fazri Labs	Android (64)		Indonesian	Tracking of symptoms (Bleeds)Social network—forumEducation	

## Role of mHealth in the Monitoring of Hemophilia Treatment

Careful documentation of bleeding episodes, infusion practices and adherence to treatment regimens are an integral part of the management of hemophilia patients, especially in the context of widely practiced home-based treatment. With the emergence of ubiquitous access to online services and mobile phones, the development of electronic applications has allowed the transition from manual treatment diaries to online tracking. While in the early 2000s electronic handheld devices were developed for electronic logging for hemophilia patients, they have not found their way into standard clinical practice. Instead, they completely disappeared with the emergence of smartphone usage (66).

mHealth might offer the potential to complement and enhance the treatment of hemophilia patients in several ways. An increasing number of different apps have been developed in mobile or web-based form for tracking of treatment regimens of hemophilia patients. Different CFC producers, as well as digital health companies have developed apps which can be used either specifically for their product or independent of the administered CFC (67). Among the currently most used and with the longest experience available apps are HemaGo by Novo Nordisk Inc., HemMobile by Pfizer Inc., FactorTrack by Bayer AG, BeatBleeds by Shire US Inc., MicroHealth Hemophilia by MicroHealth LLC or myPKFiT by Takeda Inc. (68).

## Features of Apps

The IT software developed for monitoring of treatment of hemophilia has both a patient and a health care provider's interface, each providing different data and utilization possibilities to the respective user. The patient's interface of the apps is based on the same underlying concept regardless of the specific application, equipped with slightly different features. The basic idea is reflected in an online documentation system of patient's events: infusions, compliance, and bleeds. In accordance with both FDA and EMA legislation and regulations, besides monitoring, the proper and secure documentation is an important feature of these apps. Following documentation, archiving is yet another vital function when it comes to these types of apps. This is of crucial importance in scenarios such as providing evidence in case of litigation. Length of archiving should be defined, as well as retrievability from archives—therefore, the respective responsibilities with this important function need to be unambiguously laid down. These considerations imply to exclude the manufacturers from any of these functions. With varying degrees of detail, the patient can document their bleeds, including date, duration, location, pain levels, complications, treatment administered and illustration of the lesion by pinpointing on a model, pictures, or videos. Infusions can be logged in a calendar with number of units infused and batch number (in some cases by simply scanning the bar code of the product). A reminder system will automatically notify the patient to perform infusions on previously designated days. The apps allow the patient to choose which data to share with their health care providers, either in real time or in the form of email. The health care provider's interface allows consultation of the patients' data, to the extent they decide to share it. Commonly, the health care provider is presented with a complete list of patients currently utilizing the app in an overview format. Detailed information regarding each patient can be retrieved if desired, with newer applications allowing real-life updates of the patient's data. Also, the physician can typically communicate with the patient directly through the application. Different apps have designed specific features, such as allowing patients, who are traveling or living abroad to quickly locate HTCs in different countries.

Some apps like Hemophilia Support (69, 70), have included services providing exchange between peers functioning as social networks. Others such as Hemophilia Disease (71), Hemophilia transfusion medicine (72) or Mi Hemofilia (73), offer educational programs for patients or HTC in languages other than English. Another type of app offers personalized exercise regimens for hemophilic patients (74). This case may not be appropriate as the app does not have all information on the patient and only the HTC should recommend physical exercises, tailored to each cared patient. One pharmaceutical company has developed an app specifically for its clotting factor concentrate product with the aim of personalizing the prophylactic treatment by making recommendations for dosage adjustments by estimating the patient's pharmacokinetic parameters (67). Still, we strongly believe that this function should be the exclusive privilege and responsibility of the treating physician or HTC as the PK is not the sole aspect for deciding the therapy. Yet again, some apps allow patients to sign up several family members with a single profile. All apps are free of charge for the user.

## Potential of mHealth

Mobile or web-based applications were developed with the aim of addressing certain aspects of current hemophilia treatment, as well as for marketing purposes for a specific pharmaceutical product. Still, before implementation of the apps, the elaboration of objective and critical information sheets on these specific aspects must be carried out, in order to properly raise awareness with patients to potential caveats in using the apps. Therefore, it is necessary to understand how mHealth intends to address some of the challenges encountered in current hemophilia care. Patient-Physician communication during home treatment has come with a significantly increased quality of life of the patient and has also resulted in fewer visits to medical offices and HTCs. This makes it more difficult for health care providers to monitor the incidence of bleeds, infusion practices and compliance. It might cause a delay in consultations of medical services when those are required (18). Mobile apps present an opportunity to bridge the gap between patient and physician, with real-time, two-way communication between both parties; and only between these two parties. Health care providers can monitor the patient's events and self-management from a distance and request in-person meetings at any given moment in time, if deemed necessary, to adjust the treatment regimen. The possibility to address inadequate infusion practices in a timely manner, represents an important aspect in providing the best possible care to all patients managed by the HTC (75).

Successful management of chronic disease is known to be a major challenge for patients and health care providers alike. In the treatment of common chronic diseases, such as asthma, COPD, diabetes mellitus, heart failure and hypertension, it is commonly observed that only ~50% of patients take their medications as prescribed (76).

The introduction of software-based treatment monitoring has shown promising effects on the self-management of different chronic diseases (77–79). As hemophilia is a lifelong condition whose evolution is significantly dependent on adequate prophylactic treatment, while simultaneously being plagued by the same challenges of adherence to long-term regimens, the role of mHealth must be closely considered. Two critical periods in a patient's life have been identified as being associated with a significantly increased frequency of non-adherence. Firstly, when the patient switches to self-infusion and secondly, when the patient moves away from home and must assume full responsibility of self-care (80, 81). While education remains a core aspect in addressing barriers to adherence, electronic logs, and reminder systems, constituting an integral part of apps, have been identified as a potential strategy to improve adherence (18). The speed and ease at which patients can track their treatment in apps has been cited to be an especially important feature of electronic diaries compared to paper-based diaries for hemophilia patients (82).

The patient's data collected via mobile applications could be sent to national and international hemophilia registries, only if and after being securely anonymized. This is an absolute requirement for any sharing of the data. Such sharing of personal data requires written informed consent by the patient. This serves an important purpose, as the research of hemophilia heavily relies on registries, considering the relative rarity of the disease. The small size of the patient population hampers the possibility of high quality, well-designed studies to generate the necessary evidence to evaluate current practices and provide guidelines for improvement. Currently some aspects of clinical management of hemophilia remain empirical.

While the main focus of mHealth is clearly on enhancing adherence, improving patient-physician communication, while gathering important data for registries, mHealth might also be able to address psychosocial issues experienced by patients with hemophilia. Home treatment options have inevitably led to fewer opportunities for patients to meet fellow patients at HTCs. As chronic diseases are often associated with a considerable psychosocial burden, a supportive social network of peers can play a key role in disease management. Apps equipped with social networking functions or recommendations for hemophilia-specific, restricted access social media, present interesting alternatives for building a supportive network among patients via electronic infrastructures. As providing social support to other patients has been reported by some patients as effective in reducing their own psychological burden related to hemophilia, mHealth might provide patients an opportunity to actively engage in online-based peer support systems (83). This is an alternative to engage in constructive talks and social events, especially taking into consideration the special epidemiological scenarios recently experienced with the COVID 19 pandemics. mHealth is a viable alternative to classic targeted meetings, congresses, summer or winter camps, where active presence indeed made personal contact opportunities easier.

## Current Research and Challenges

Research evaluating the use of mHealth in the management of hemophilia patients has evolved gradually over the last two decades. Studies conducted thus far have mostly aimed to investigate and understand the impact of mHealth on record keeping, compliance to prophylactic treatment, quality of life, communication between patient and physician and joint damage (58, 82, 84). Several studies only examined the effect of one specific electronic application on hemophilia management (59, 75, 85). Further, the period that was evaluated of patients utilizing electronic tools was relatively short, ranging from 3 months to 1 year, considering that prophylactic treatment should be performed lifelong. The average sample size of conducted studies was comparatively small, ranging from 20 to 99 patients (84, 86). Current research concluded that mHealth seems to improve the health-related quality of life of the patient, as well as the perception of the illness (58). It might also improve communication between physician and patient (82). While it may seem that record keeping and adherence might improve within the period of the conducted research, this finding requires further investigation, as such a short period of time as chosen by most studies, will not be sufficient to quantify the long-term effect on compliance. Apart from already published studies, additional research projects could potentially investigate one interesting aspect with the use of mHealth: the impact on the business of pharmaceutical companies by these devices.

Thus far, it was not observed that mHealth has a significant effect on long-term quantifiable health outcomes such as joint health (87). These results are surprising if the presumptions are correct that prophylactic treatment protects the joints of the patients and that mHealth increases compliance of patients on regular infusions to keep the trough-levels up. A survey from 2017 aimed to establish a picture of current mHealth usage in the hemophilia community regardless of specific app, understand the reasons for non-use or discontinuation of mHealth related to both patient and physician factors and possible challenges to implementation of apps on a large scale. Among the identified factors for lack of use of apps, were cost of apps, concerns over privacy and safe storage of patient data, lack of regulatory approval, perceived level of difficulty of understanding the app and general lack of evidence regarding its efficacy in general clinical practice. Privacy code of conduct of mHealth should promote trust between users and help patients with a better therapeutic outcome. Initially, the first versions for the code of conduct were prepared against the background of the 2014 mobile health green paper consultation of the European Commission. And the later consultation indeed showed that users did not trust mHealth apps because due to privacy concerns. Thus, the Commission strongly encouraged the industry to promote a privacy code of conduct that would increase trust, that would obtain its approval. Under the General Data Protection Regulation (GDPR), this objective fell under the authority of the European Data protection Board, as codes approved by it are granted validity across the European Union through an implementing act. The mHealth code of conduct was drafted by a team of industry members with the European Commission acting as a facilitator, that provided policy expertise and resources, a team that included the App Association (ACT), App developers Alliance, Apple, COCIR, Digital Europe, ECHA, DHACA, EFPIA, Google, Intel, Microsoft, Qualcomm, and Samsung.

The code was submitted in December 2017, with the approval of the Data Protection Directive and addressed important topics, such as user content, that stated that “user consent for the processing of personal data must be free, specific and informed. Explicit consent needs to be obtained for the processing of health data. Any withdrawal of consent must result in the deletion of the user's personal data.” For the purpose of data use minimization, it stated that data could only be used for specific and legitimate purposes and only data required for functionality was to be used. Privacy is intended to be ensured by design and by default and its implications should be considered at each step of development. Regarding access to personal data, the user is entitled to access the data and request corrections, should it be necessary. Personal data should not be stored if not compulsory. Regarding safety, technical measures must be implemented to ensure confidentiality, integrity, and availability, to protect the user from accidental or unlawful destruction, loss, alteration, disclosure, access, or other unlawful forms of processing. Data processing for secondary purposes must be compatible with the original one and further processing, for either scientific or legislative purposes for non-compatible purposes needs a second signed informed consent. For data transfers to a location outside the European Union/ European Economical Area, there needs to be legal guarantees permitting such transfer, as is an adequacy decision of the European Commission, European Commission Model Contracts or Binding Corporate Rules.

These are key issues because they are of paramount importance when it comes to legal and practical considerations. The survey failed to provide statistically significant results. However, it might serve as a starting point for further research questions, as it has focused the attention on issues previously not evaluated by another research (68). Still, mHealth applications without adequate access to products and care is of limited value, as is the case of Romania so far.

Challenges associated with mHealth as part of monitoring of treatment in hemophilia patients are mainly related to lack of data and understanding of this new tool, while a second aspect is mainly related to problems associated with the technology itself. As the use of IT software in the monitoring of hemophilia patients is a relatively new component of disease management, it is naturally characterized by a lack of enough data, identifying its exact significance, role, and associated issues. The validity of current research in this area is limited by small sample size and study design. Enough high-quality evidence supporting the effectiveness of apps, as claimed by some product designers, is still lacking. As research results on mHealth in hemophilia management in general, regardless of specific app, are scarce, recommendation for general clinical practice can only be made on limited grounds.

Further, the proportion of patients using electronic applications has not been extensively quantified and will also be difficult to compare them as several of the listed apps are offered by competing CFC-manufacturing companies. The reasons for lack of usage or discontinuation of apps remain largely unknown. Also, evaluation of long-term impact of mHealth on hemophilia management has not be sufficiently evaluated yet, as the technology is still too modern. Lastly, within the current discussion of mHealth in treatment monitoring of hemophilia patients, there is a blind spot related to the education of physicians on the adequate usage of electronic tools. If physicians cannot appropriately familiarize themselves with this aspect of treatment, it will be unlikely that they will recommend it to their patients, inhibiting further implementation.

A second major aspect that requires consideration in this context, are the problems related to web-based and mobile phone technology itself. While the younger generation, as digital natives, typically has little issues embracing a new application, it is important to understand the challenges that older patients and physicians might face when meeting them. More intensive education on the apps might be required to be sufficiently understood. Further, it is important to provide patients with an application design that ensures that their data will be safely transferred and stored, as digital safety has been identified as a concern by many patients (46) and experts (65). What is really of key importance is the secure and authorized handling of private and confidential data. In analyzing the development and privacy in data ownership in mobile health technologies in the United States, Galvin and DeMuro concluded that mHealth data storage and transmission is of crucial importance, as such data was previously “de-identified” and now demonstrated to be “re-identifiable” (88). The international regulatory framework that serves to protect human data is diverse and the bog challenges for regulators is to keep up with rapidly advancing technology to serve the best interests of patients and their caregivers (89–92).

As certain pharmaceutical companies have signed apps specifically for their respective product, it might leave the patient unable to import data from one app to another if they need to change their treatment. Lastly, app developers should keep in mind possible incompatibilities between apps and certain mobile phone models, restricted access to apps in certain geographical areas or inability to localize apps in the app store.

## Real-Life Setting in Romania in 2021

One of the three priorities of the Europe 2020 strategy is smart growth, by developing a knowledge-and-innovation-based economy. Romania responds to this strategy by pursuing the development of human resources to provide further services at a higher professional level and by introducing modern technologies and innovative solutions in the regional areas of strategic importance in Romania: South (Bucharest), West (Timisoara), North-East (Iasi) and North-West (Cluj) Development Regions. In hemophilia management, Romania has contributed in the last 3–5 years to the priority of sustainable growth, as through training, internal staff use more efficiently the available resources and equipment, leading, in the long term, to increasing the competence of the medical act. In 2021 in Romania, while people with hemophilia can lead fairly normal lives, certain precautions to prevent and control bleeds, managing the condition can be challenging. In 2020, the actual rate of FVIII consumption was of 3.42 IU/capita/year and that of FIX was 0.2 IU/capita/year, slightly lower than that of 4 IU/capita/year and 0.5 IU/capita/year for FVIII and FIX, respectively, as recommended by the European Council. Those living with hemophilia or caring for someone with hemophilia can face a wide range of difficulties—including medical, psychological, social, and financial—which is why a strong network of support is a vital part of comprehensive care. It is estimated that 500 people in Romania are annually diagnosed with hemophilia. Despite the advantages of treating the bleeding episodes early, significant barriers and limitations remain. The most important barrier is the educational barrier, which involves lack of awareness among patients regarding the signs of a bleed, as well as importance of early therapy. It is also common for parents or caregivers of school-age children to exhibit inconvenience and scheduling barriers. Distance to the treatment center can also play a role here. Some patients experience financial barriers related to cost of clotting factor products, insurance coverage, or insurance caps and out-of-pocket costs. Rarely, there can also be problems related to venous access or home infusion. Lastly, multiple psychosocial barriers can prevent adherence to treatment regimens. Identification and addressing these individual barriers, especially by the patient communities and the Romanian Society of Hematology, have resulted in improved compliance rates, prevent joint damage, be more cost-effective, and lead to better overall health of these patients. The common challenges are similar in other neighboring countries, also with low levels of levels of economic and social development (Serbia, Bulgaria, and Republic of Moldova). Targeted research shows that the incidence of hemophilia is growing in less developed countries considering the existing gaps in access to treatment in relation to the western nation, where access to training and high-tech equipment is easily available. In the past, insufficient technical equipment that contributes to health inequalities—regional/local (disadvantaged) communities' reduced access to an accurate laboratory diagnosis and disease status. These challenges have been addressed, with the development of proper diagnosis and monitoring by specialized laboratories, as well as with the introduction and functioning of a national registry of hemophilia and rare coagulation disorders. As Romania is one of the emerging countries regarding IT technology, mHealth comes naturally and introduces new alternatives for hemophilia monitoring, especially in the younger population. Still, mHealth applications in the context of access to care, is of limited value, taking into consideration that Romania has an aging population.

## Conclusions

The World Federation of Hemophilia acknowledged the potential of using software-based logging in the management of hemophilia patients, in the 3rd edition of their guidelines, published in 2020. It was recognized that use of electronic logging has demonstrated an increase of information provided, as well as completeness of data reporting. Also, it is suggested that the use of electronic tracking might improve the patient's quality of life, support HTCs in modifying treatment regimens and improve communication with the healthcare team. The advent of mHealth can be considered an important step in hemophilia care, containing within itself the opportunity to improve patient management, quality of life and long-term health outcomes overall. The exact role of mHealth in hemophilia management remains to be determined in following years to come, as further understanding of the currently promising advantages and potential risks of electronic tools needs to be gained. Education of health care professionals regarding mHealth remains another issue that needs to be considered. In order to overcome existing challenges to mHealth use among patients with hemophilia, it might be advisable to involve patients more extensively and intensively in the development of treatment monitoring methods, as they can input their expertise and experience; and might be able to contribute a wealth of information in order to further advance certain aspects of hemophilia management.

## Author Contributions

All authors have contributed to the design and writing of the manuscript.

## Conflict of Interest

MSt was employed by Takeda Pharmaceutical Company. The remaining authors declare that the research was conducted in the absence of any commercial or financial relationships that could be considered as a potential conflict of interest.

## Publisher's Note

All claims expressed in this article are solely those of the authors and do not necessarily represent those of their affiliated organizations, or those of the publisher, the editors and the reviewers. Any product that may be evaluated in this article, or claim that may be made by its manufacturer, is not guaranteed or endorsed by the publisher.
